# Combined effects of recent Pacific cooling and Indian Ocean warming on the Asian monsoon

**DOI:** 10.1038/ncomms9854

**Published:** 2015-11-13

**Authors:** Hiroaki Ueda, Youichi Kamae, Masamitsu Hayasaki, Akio Kitoh, Shigeru Watanabe, Yurisa Miki, Atsuki Kumai

**Affiliations:** 1 Faculty of Life and Environmental Sciences, University of Tsukuba, 1-1-1 Tennodai, Tsukuba, Ibaraki 305-8572, Japan; 2 Center for Global Environmental Research, National Institute for Environmental Studies, 16-2 Onogawa, Tsukuba, Ibaraki 305-8506, Japan; 3 Graduate School of Life and Environmental Sciences, University of Tsukuba, 1-1-1 Tennodai, Tsukuba, Ibaraki 305-8572, Japan

## Abstract

Recent research indicates that the cooling trend in the tropical Pacific Ocean over the past 15 years underlies the contemporaneous hiatus in global mean temperature increase. During the hiatus, the tropical Pacific Ocean displays a La Niña-like cooling pattern while sea surface temperature (SST) in the Indian Ocean has continued to increase. This SST pattern differs from the well-known La Niña-induced basin-wide cooling across the Indian Ocean on the interannual timescale. Here, based on model experiments, we show that the SST pattern during the hiatus explains pronounced regional anomalies of rainfall in the Asian monsoon region and thermodynamic effects due to specific humidity change are secondary. Specifically, Indo-Pacific SST anomalies cause convection to intensify over the tropical western Pacific, which in turn suppresses rainfall in mid-latitude East Asia through atmospheric teleconnection. Overall, the tropical Pacific SST effect opposes and is greater than the Indian Ocean SST effect.

## Abstract (web summary)

Unique cooling in the tropical Pacific and warming in the Indian Ocean over the past 15 years is postulated to have an effect on Asian rainfall. Here, based on a numerical modelling experiment, the authors investigate this relationship and provide insight into the atmospheric dynamics at play.

## Graphical abstract

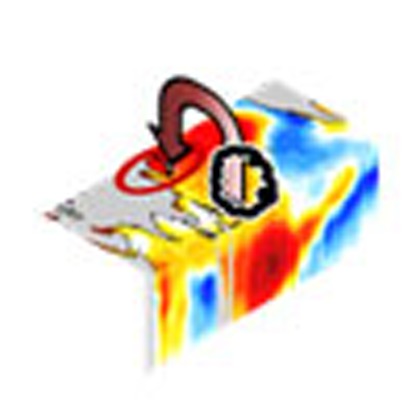

The Asian summer monsoon is a vital source of water for the world's most densely populated regions, and it underpins the livelihoods and economic activities of nearly half of the global population. Consequently, changes in monsoon rainfall are of great scientific and societal importance. Future climate projections suggest that the global monsoon precipitation is enhanced because of increasing water vapour^1^,^2^,^3^ (hereafter referred to as thermodynamic effects) according to global warming. Observed global monsoon precipitation on land decreased between the 1940s and 1980s (refs 4, 5), and showed an increasing tendency until the beginning of this century^6^,^7^,^8^. In contrast, South Asian monsoon precipitation has not followed this recent increasing trend^9^. Such regional differences in monsoons are caused in part by the effect of anthropogenic aerosols, which suppress local precipitation^10^. In addition, recent monsoon studies indicate that change in atmospheric vertical motions (hereafter referred to as dynamic effects) associated with circulation teleconnection explain the observed contrast between increasingly dry and wet climates^11^, and thermodynamic effects^1^,^2^,^3^,^12^,^13^ are secondary. For example, summertime rainfall over Northeast Asia decreases since the late 1980s (ref. 14), although the global mean temperature and local area-averaged temperature have steadily risen since the 1950s (ref. 15), implying an importance of atmospheric circulation change.

During the first decade of the twentyfirst century, the rate of increase in global average surface temperature has slowed in comparison with those in the preceding decades^16^,^17^. Our understanding of the current hiatus has recently progressed markedly^18^,^19^,^20^,^21^,^22^,^23^. La Niña-like sea surface temperature (SST) anomalies (hereafter referred to as LNSST) prevailed over the tropical Pacific and contribute to the global warming hiatus, together with external forcings such as solar irradiance^24^ and volcanism^25^. The prolonged LNSST period corresponds to the negative phase of the Pacific Decadal Oscillation, and is associated with an intensified Walker circulation across the tropical Pacific^26^,^27^. The recent slowdown of global warming is associated with pronounced regional anomalies. Specifically, the prolonged droughts over the Southern US and California^28^ have been tied to the Pacific LNSST pattern. It is unclear what changes have occurred in the Asian monsoon and to what extent they are due to the hiatus and decadal SST anomaly.

## Results

### Observed change in rainfall and sea water temperature

**Figure 1:**
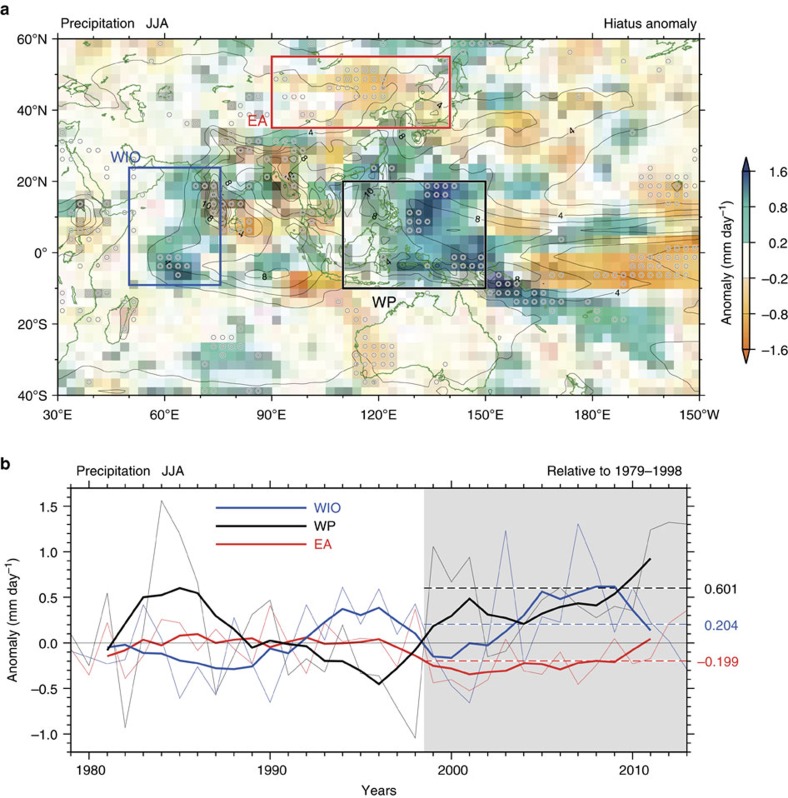
Observed change in precipitation in the Indo-Pacific region for JJA. (**a**) Regime differences between the periods 1999–2013 and 1979–1998 for precipitation. Areas with a confidence level of >95% are denoted by open circles. Thin solid contours denote climatological (1979–1998 mean) precipitation. (**b**) Time series of the mean precipitation relative to the base period (1979–1998) averaged over the EA, WIO and WP regions. Bold lines are 5-year running means, and dashed lines represent the mean values over the hiatus period (1999–2013). JJA: June, July, August.

We investigate influence of the hiatus on the Asian monsoon. The regime difference between pre-hiatus (1979–1998) and hiatus (1999–2013) periods shows that summer rainfall^29^ increased greatly over the western Pacific (WP), but decreased over middle to high latitude East Asia (EA) (Fig. 1a). Specifically, the observed change in vertical motion over the EA shows enhancing subsidence (Supplementary Fig. 1), which is consistent with *in situ* less rainfall, suggesting modulation of large-scale circulation. We will return this issue later in the numerical experiment. Noticeable positive rainfall anomalies also occurred over the western Indian Ocean (WIO), probably associated with Indian Ocean warming^30^,^31^. Although the warming covered in the entire Indian Ocean, positive and negative rainfall anomalies coexist in the eastern Indian Ocean. A significance test for employing Welch's statistics confirmed that those increasing and decreasing tendencies in the recent decadal variation in the regional rainfall are significant at the 5% level.

These observations suggest that other mechanisms are at work beyond simple thermodynamic effect of precipitation change due to Clausius–Clapeyron-related increase in specific humidity. Here we examine these mechanisms by numerical experiments. To determine whether the recent rainfall anomalies are due to the artefact of a short-lived event, we plot in Fig. 1b the time series of rainfall anomalies over three key regions, which are defined in this paper as follows: the WP (10°S–20°N, 110°–150°E), EA (35°–55°N, 90°–140°E) and the WIO (10°S–25°N, 50°–75°E). In comparison with the pre-hiatus period, rainfall increased during the hiatus period in the WP region (0.6 mm day^−1^; 10% increase relative to the pre-hiatus period) and the WIO region (0.2 mm day^−1^; 7% increase), while the EA region is characterized by prolonged negative anomalies (−0.2 mm day^−1^; 6% decrease). These changes are also recognizable in other rainfall data (see Methods). Interestingly, the running averages of rainfall in the WP and WIO are out of phase before the hiatus period; however, their trends are in phase from the late 1990s. The other notable feature is that the EA rainfall shows no salient anomalies during 1979–1998, followed by a significant decrease during the hiatus period. The decadal rainfall variation can also be found in long-lasting observational network (since 1951) over Northeast China^14^. These variations suggest a complex and changing relationship among regional rainfall indices, which may be due to the evolution of decadal SST anomalies. We investigate remote SST forcing using sensitivity experiments.

**Figure 2:**
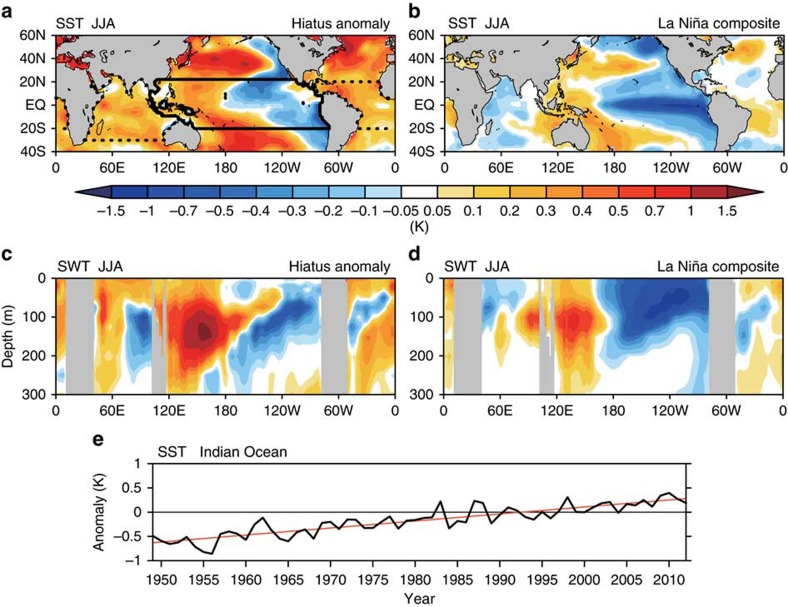
Global anomalies of sea water temperature in the boreal summer during the hiatus period compared with typical La Niña years. (**a**) Observed SST anomalies for 1999–2013 minus 1979−1998. Regions of the Indian Ocean, the tropical Pacific Ocean and the tropical Atlantic Ocean bounded by thick lines correspond to the IO, TPO and ATL simulations, respectively (see Methods). (**b**) Composite SST anomalies for La Niña years (1950, 1954, 1955, 1964, 1970, 1971, 1973, 1975, 1984, 1985, 1988, 1995 and 1998) relative to the base period (1950–1998). (**c**) Longitude-depth section of SWT anomalies along the equator (5°S–5°N) during the hiatus period. (**d**) As for **c**, but for La Niña years. (**e**) Time series of SST anomalies from the base period (1979–1998) in the tropical Indian Ocean (30°S–20°N, 40°–120°E). Red line shows a linear trend (0.145 K decade^−1^).

Figure 2a,c shows anomalies of SST and the underlying sea water temperature (SWT) during the hiatus period on the basis of historical ocean temperature analysis^32^. The SST patterns in the tropical Pacific Ocean (TPO) resemble the LNSST-related negative phase of the Pacific Decadal Oscillation. This indicates that natural variability accounts considerably for the observed hiatus LNSST^22^ rather than anthropogenic forcing (Supplementary Fig. 2, Supplementary Table 1 and Supplementary Note 1). A close relationship between the TPO and the Indian Ocean is widely recognized on the interannual timescale. In the post El Niño summer, while positive SST anomalies in the eastern Pacific vanish, or become slightly negative, positive SST anomalies persist over the tropical Indian Ocean^33^. The reverse is true for the tropical Indian Ocean during summer following La Niña with occupied by cold water (Fig. 2b,d). It is interesting to note that the entire Indian Ocean has continued to warm^30^,^31^ since the 1950s (Fig. 2e). The SST pattern is inconsistent with the interannual relationship with La Niña-like cold SST anomalies in the TPO. In other words, the combination of the warming Indian Ocean and the Pacific LNSST is unprecedented with potentially large impact on the Asian monsoon. A number of recent studies have drawn attention to interannual as well as interdecadal variability in the Pacific Ocean and its influence on the Asian summer monsoon. Statistically, the Asian summer monsoon tends to be intensified during the La Niña condition^34^ and/or negative phase of the PDO (La Niña-like anomaly)^35^. Having noted these processes, we expect that enhancement of the Asian summer monsoon during the hiatus period. However, the observed rainfall distribution exhibits non-homogeneous pattern in the Indo-WP (Fig. 1a), which requires other mechanisms than the Pacific forcing.

Pacific SWT anomalies (Fig. 2c), positive in the WP and negative in the central to eastern Pacific, are consistent with the Pacific LNSST. Meanwhile, the mixed layer (*ca.* 50 m) of the tropical Indian Ocean is occupied by warm water, and the SWT anomalies around the thermocline (50–100 m) exhibit patterns similar to the Indian Ocean zonal dipole mode^36^ that tends to occur during El Niño events. The SWT pattern features the basin-wide surface warming and the Indian Ocean dipole-like subsurface anomalies are worth further investigation. Here we focus on the SST anomalies in the tropical Indo-Pacific and elucidate their effects on recent changes in rainfall over the Asian continent and surrounding oceans.

### Sensitivity experiments to the decadal SST anomalies

Recent study suggests that the internal climate variability is important in the rainfall variation in the mid-latitude when they use atmosphere–ocean-coupled model with different initial conditions^37^,^38^. Whereas if we use atmospheric model forced with fixed SST, resultant spread of atmospheric teleconnection, obtained from multimodel ensemble as well as initial condition ensemble, becomes smaller^39^,^40^. The observed rainfall over EA shows a large interannual variability, indicating an importance of internal climate variability. In addition to the interannual variation, large decadal-to-interdecadal variation can also be found in the observed EA rainfall^14^ (Fig. 1b), which requires alternative explanation other than the mid-latitude internal climate variability. The aim of the present paper is to examine the influence of unique patterns of decadal SST anomalies during the hiatus period on circulation change. Therefore, we carried out 10-member ensemble sensitivity experiments using the Meteorological Research Institute atmospheric general circulation model^41^ (MRI-AGCM; see Methods). In our simulation, the global SST anomalies during the hiatus period, including the LNSST pattern in the Pacific and warm anomalies over the entire Indian Ocean (Fig. 2a), are prescribed.

**Figure 3:**
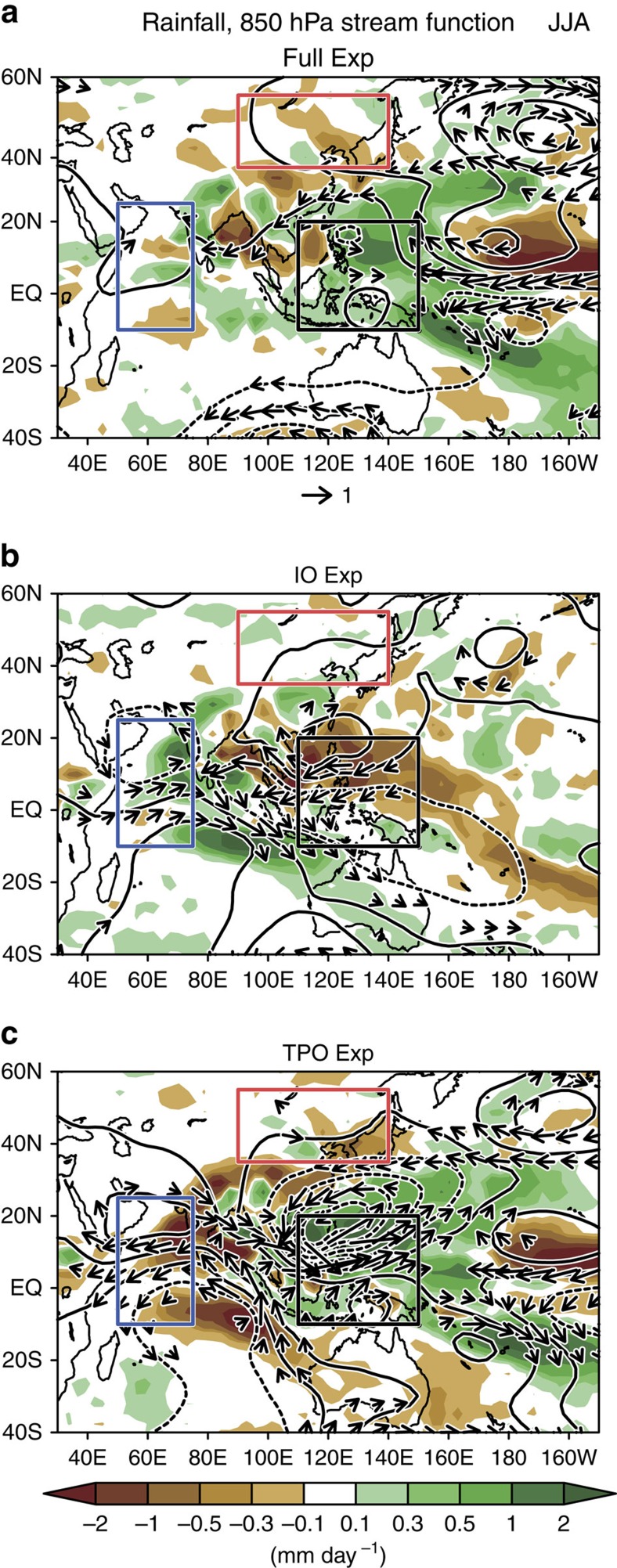
Simulated patterns of circulation in the Indo-Pacific region for the boreal summer. (**a**) Precipitation (mm day^−1^), wind vectors (m s^−1^) and stream function (10^6^ m^2^ s^−1^) at 850 hPa from MRI-AGCM 10-ensemble experiments, where SSTs are set to hiatus anomalies (1999–2013). (**b**,**c**) As for **a**, but for the effect of the Indian Ocean and tropical Pacific, respectively (see Methods). Red, blue and black rectangles show the EA, WIO and WP regions, respectively.

As shown in Fig. 3a (referred to as Full Exp), in response to these SST anomalies, the MRI-AGCM qualitatively reproduced the observed decadal negative anomalies in EA rainfall (Fig. 1a). The statistical analysis for our simulation confirms that the rainfall anomalies over the EA and WP are statistically significant at 95% confidence level (Supplementary Fig. 3). We note that the WIO rainfall change is too weak in the Full experiment (Fig. 3a) in comparison with those in the observations (Fig. 1a). As for the wind field in Full Exp, the intensified tropical easterlies and anomalous cyclonic circulation around Philippines are consistent with the observations (Supplementary Fig. 1). These features are also indicative of strengthening of the Walker circulation together with intensified convection over the WP.

Next, we conducted a series of AGCM experiments that prescribed SST forcing in the Indian Ocean (referred to as IO Exp), TPO (referred to as TPO Exp) and Atlantic Ocean (referred to as ATL Exp) to isolate their influence on the circulation fields during boreal summer. In the IO Exp (Fig. 3b), rainfall increases over the Indian Ocean and decreases over the northwest Pacific near the Philippines with an anomalous anticyclonic circulation. In general, enhanced tropical convection together with upward motion causes subsidence over the rest of the tropics. Specifically, localized subsidence can be ascribed to atmospheric response to the heating anomaly. By the use of a linear baroclinic model, Kelvin-wave-induced easterly anomalies emerge over the subtropical Pacific along ∼10°N due to an anomalous heating over the tropical Indian Ocean^33^,^42^. This in turn led to a strengthening of the anticyclone over the northwest Pacific centred around 20°N, 120°E, which closely resembles the anomalous anticyclone in our IO Exp.

In the TPO Exp (Fig. 3c), salient rainfall increase in the WP is recognizable, which may be caused by deep warming in the WP throughout the thermocline (Fig. 2a,c). On the other hand, rainfall decreases in the WIO region and the mid-latitude EA region around the northern edge of the enhanced convection in the WP. The latter is physically consistent with Pacific–Japan teleconnection^43^,^44^. The reduction of rainfall in this region, accompanied by the enhanced rainfall around Philippines, is smaller than that in Yangtze River Basin (around 30°N) but is statistically significant^44^ (Fig. 3c). As for the tropical Indian Ocean, the Pacific-origin anomalous twin anticyclones emerge across the equator (Fig. 3c), causing strong easterlies. The simulated wind is consistent with typical features of the atmospheric response to the imposed heating relevant to the Pacific LNSST^13^. The opposing wind anomalies in the tropical Indian Ocean between the IO Exp and the TPO Exp are responsible for the aforementioned weak wind anomalies in the Full experiment. The simulated rainfall anomalies bear resemblance with those in the past similar SST experiments. During 1998–2002 after turnabout of the 1997/98 El Niño, corresponding to warm anomalies in Indo-WP, salient increase in the tropical rainfall strengthens subsidence in the rest of adiabatic heating region^45^. In addition to this, cold SST anomalies in the central–eastern Pacific spanning the same period has synergy impacts on a drying pattern over the mid-latitude including the United States and Southwest Asia^46^. Our study examines the isolated impact of the warmed Indian Ocean, which provides additional insight about inherent regulating mechanism for the rainfall distribution.

Table 1 is a summary of the impacts of SST anomalies in each ocean on rainfall change in the three key regions. The TPO effects on WP as well as WIO are two to three times greater than those of IO, and with opposite signs. In the most cases, the ATL experiment exhibits similar to the IO Exp, acting in the direction cancelling nonlinearities for the Full Exp. The contribution of the Atlantic Ocean to the rainfall anomalies is slightly smaller than those of IO with significant negative rainfall anomalies in WP. This response is consistent with warm SST anomalies in the Atlantic Ocean (Fig. 2a) and a resultant decrease in rainfall over the WP through modulation of the Walker circulation^47^. These results indicate that the rainfall changes should be ascribed not only to local SST anomalies but to remote SST-induced opposing effect. Hereafter, we focus on the IO and TPO effect because the ATL effect is relatively smaller than other effect.

**Figure 4:**
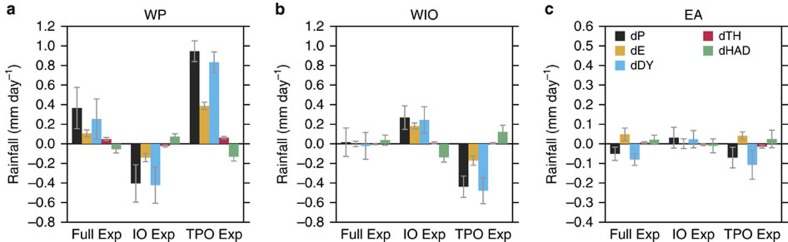
Vertically integrated moisture budget incorporating tropical Pacific and Indian Ocean effects. (**a**) Dynamic effect (dDY), thermodynamic effect (dTH), horizontal advection (dHAD) and evaporation (dE) averaged over the WP region against for the net precipitation change (dP) based on 10-ensemble experiments. (**b**) As in **a**, but for the WIO region. (**c**) As in **a**, but for the EA region; see Methods. Note that the vertical scale of **c** is half that of **a**,**b**. The 5% significant level obtained from the Student's *t*-test is denoted by error bars.

The spatial distributions of decreased/increased rainfall in Fig. 3 correspond well with downward/upward motion (Supplementary Fig. 4), indicating an importance of dynamic effect on the rainfall change. Therefore, to improve our quantitative understanding of the rainfall variation, we calculated vertically integrated moisture budgets^12^ (see Methods) and decomposed the dynamic and thermodynamic processes associated with the rainfall variations over the three regions (Fig. 4). Note that linear additivity does not hold because we do not consider effects from the extratropics. However, as shown in Table 1, the TPO and IO effects are dominant players in the total rainfall change. In the WP region (Fig. 4a), the net effect of the full SST anomaly during the hiatus (Full Exp; black bar) is to enhance rainfall, which is associated with the dynamic effect (upward motion) and enhanced evaporation from the sea surface. The rainfall increase is closely related with the tropical Pacific (TPO Exp); however, the subsidence induced by Indian Ocean warming corresponds to the WP rainfall decrease, and this is manifested in a negative dynamic effect in the IO Exp. Over the WIO (Fig. 4b), the full SST effect is almost zero. The rainfall change associated with the Indian Ocean warming is largely offset by the remote influence of dynamic effect originating from the tropical Pacific SST (see also Fig. 3). These results indicate that in both the Indian Ocean and WP the Pacific LNSST anomalies and the Indian Ocean warming tend to oppose each other in the atmospheric moisture budget. In the most cases, rainfall change over the WP in response to the local SST anomaly is larger than those over the Indian Ocean. Over the EA region (Fig. 4c), the negative dynamic effect originating from the LNSST anomalies in the tropical Pacific is associated with the net decrease in rainfall despite increased evaporation in the WP. The effect of Indian Ocean on EA is small. This is consistent with the position of anomalous anticyclone (Fig. 3b), dominating to the south of EA region. S.d. among 10 members (Table 1) is large in the EA region, indicating an importance of internal climate variability over the mid-latitude. Significant model biases found in Full Exp (for example, large positive rainfall in 110°E, 40°N that is not found in the observation) contribute to decrease the area-averaged rainfall anomalies. However, most of water budget components including dP except WIO rainfall in Full Exp and EA rainfall in IO Exp are statistically significant at 95% confidence level in the three key regions.

## Discussion

Over the mid-latitude, internal climate variability is of great importance in rainfall variations on interannual-to-interdecadal timescales^37^,^38^. As a matter of fact, Figs 3a and 4 and Supplementary Fig. 3a show that the atmospheric internal variability has a significant role in rainfall variations over the mid-latitude EA. In the mid-latitude EA, the spread is comparable to the ensemble mean anomalies (Table 1), suggesting an importance of atmospheric variability on precipitation change. However, if the atmospheric internal variability is dominant player in the rainfall change in the mid-latitude, the rainfall anomaly can be either positive or negative. Whereas the time series of rainfall during the hiatus period (red line in Fig. 1b) exhibits continued less rainfall since the late 1990s, which requires alternative explanation other than the internal variability. Given these results, it is conceivable that the rainfall decrease over the mid-latitude EA during 1999–2013 could be results from combination of the atmospheric internal variability and the WP-origin atmospheric teleconnection^43^,^44^.

Our results show that the Indian Ocean warming has a dynamic suppressing effect on convection in the WP region; however, the opposing effect from the anomalous tropical Pacific SST is greater, resulting in a net rainfall increase. As LNSST conditions dissipate in the near future^21^ and the Indian Ocean continues to warm in the coming decades, how will this new combination of SST affect the Asian monsoon? Our results suggest that, with increased SST over the both Indian and Pacific Oceans, convection weakens over the WP with a dynamic effect to increase rainfall in the EA region. Similarly, rainfall in the WIO region would increase through the dynamic teleconnection from the Pacific together with enhanced evaporation from the local ocean warming.

Our findings are important in understanding rainfall changes under a CO_2_-enriched atmosphere. Most climate models in the Coupled Model Intercomparison Project phases 5 and 3 show a projected increase in rainfall over the EA region^48^ due to water vapour increase^2^,^3^. The projected increase in summer monsoon rainfall over the ocean generally corresponds to the underlying warmed SST and the resultant increase in moisture convergence^2^,^3^,^12^. Recent studies indicated that rainfall changes under global warming condition exhibit regionally different response associated with shifts in convergence zone due to spatial gradient of SST warming^49^,^50^,^51^,^52^. Our study highlights the need to consider dynamic teleconnection through interbasin SST forcing to understand regional and decadal-to-interdecadal rainfall variations. The slow SST processes offer potential predictability for the populous Asian monsoon region.

## Methods

### Observational data sets

For our analysis of climate variation in the atmosphere and ocean, we used the following: (1) monthly precipitation data from the Global Precipitation Climatology Project version 2.2 on a 2.5° × 2.5° grid^29^ for the period 1979–2013 and (2) SST and SWT data archived by the Japan Meteorological Agency for 1950–2013 (ref. 32) as a global ocean heat content data set on a 1° × 1° grid with 23 vertical layers from 0 to 1,500 m depth. Regarding the time series of rainfall in the three key regions, similar results were obtained from CPC Merged Analysis of Precipitation^53^. To check the circulation anomalies, we used ERA interim^54^ for winds at 850 hPa and vertical motion (omega) at 500 hPa (Supplementary Fig. 1).

### AGCM sensitivity experiments

We examined the influences of the SST anomalies on the Asian summer rainfall by ensemble model simulations using an atmospheric general circulation model (AGCM), MRI-AGCM^41^. The resolution is T42 spectral truncation (horizontal spacing of ∼300 km) and 30 vertical layers. Four 10-member ensemble simulations (namely, Pre-Hiatus, Hiatus, IO Exp and TPO Exp) were conducted to decompose the influences of SST anomalies over the IO and TPO (Fig. 2a). Influence of the Atlantic Ocean (ATL Exp) was also examined by three-member ensemble simulations. By using ERSST v3 (ref. 55), 10-year integration for Pre-Hiatus run prescribed with climatological monthly mean SST averaged for 1979–1998 was conducted and the last 9 years were used for the analyses. Hiatus run was conducted in a similar way but prescribed with SST averaged for 1999–2013. SSTs prescribed in IO and TPO runs were as the Pre-Hiatus run except in the IO and TPO regions (thick lines in Fig. 2a), respectively, where the Hiatus values were used. Influences of the global-SST, IO-SST and TPO-SST during the hiatus period were estimated by Hiatus minus Pre-Hiatus, IO minus Pre-Hiatus and TPO minus Pre-Hiatus, respectively. We also examined the two SST effects (IO and TPO SST anomalies during the hiatus period) by comparing Hiatus run and other sensitivity runs (prescribed SST is identical to Hiatus run but is replaced by that in Pre-Hiatus run over IO and TPO regions, respectively) and confirmed that estimated effects of IO and TPO SST were similar to those shown in this study.

### Moisture budget analysis

The mean precipitation changes for the months June–August can be decomposed into dynamic and thermodynamic components, corresponding to the respective effects of atmospheric circulation and water vapour. For a detailed evaluation in conjunction with three-dimensional (3D) motion, we performed a column-integrated water budget analysis using the following equation^12^:


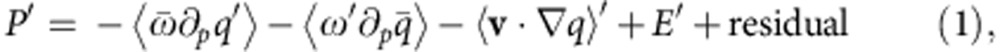


where the operator ^−^ denotes the Pre-Hiatus value and *'* represents a deviation from the Pre-Hiatus value. Here *P*, *ω*, *q* and *E* are precipitation, vertical *p*-velocity, specific humidity and evaporation, respectively. The operator 〈 〉 represents vertical mass integration through the troposphere. The right-hand terms represent the ‘thermodynamic effect' associated with changes in water vapour, and the ‘dynamical effect' associated with changes in vertical velocity, as well as horizontal advection, evaporation and residual.

### Code availability

The atmospheric general circulation model adopted here is version 2.3 of the Meteorological Research Institute (MRI), which is available under collaborative framework between MRI and related institute or university. The data from this paper are accessible via the authors.

## Additional information

**How to cite this article:** Ueda, H. *et al.* Combined effects of recent Pacific cooling and Indian Ocean warming on the Asian monsoon. *Nat. Commun.* 6:8854 doi: 10.1038/ncomms9854 (2015).

## Supplementary Material

Supplementary Information Supplementary Figures 1-4, Supplementary Table 1, Supplementary Note 1 and Supplementary References.

## Figures and Tables

**Table 1:** Influence of SST anomalies on rainfall change.

**Rainfall (mm per day)**	**WP**	**WIO**	**EA**
Full Exp	0.37±0.26	0.02±0.18	−0.05±0.04
IO Exp	−0.41±0.24	0.27±0.15	0.03±0.07
TPO Exp	0.95±0.13	−0.44±0.14	−0.07±0.10
ATL Exp	−0.18±0.08	0.06±0.07	0.03±0.08

ATL, Atlantic Ocean; Exp, Experiment; IO, Indian Ocean; SST, sea surface temperature; TPO, tropical Pacific Ocean; WIO, western Indian Ocean; WP, western Pacific.

One s.d. (second number) is computed based on 10-member (Full, IO and TPO) and three-member ensemble (ATL).
