# Supplementary material for: Combined effects of recent Pacific cooling and Indian Ocean warming on the Asian monsoon
Source: Nat Commun. 2015 Nov 13;6:8854. doi: 10.1038/ncomms9854 (PMC4660201; doi:10.1038/ncomms9854)
Supplement: Supplementary Information — Supplementary Figures 1-4, Supplementary Table 1, Supplementary Note 1 and Supplementary References. [file ncomms9854-s1.pdf]

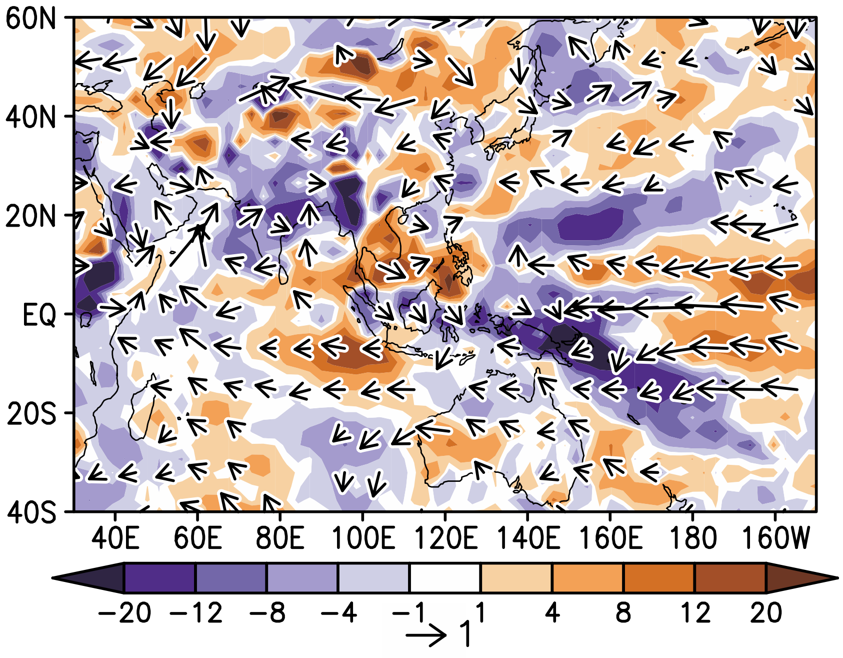

Supplementary Figure 1 | Observed change in wind and vertical motion. Anomalies are regime differences between periods 1999–2013 and 1979–1998 obtained from ERA-interim. Vectors are horizontal wind at 850 hPa level (m s^{-1}). Shadings denote omega (hPa day^{-1}) at 500 hPa. Positive (negative) values indicate downward (upward) motion.

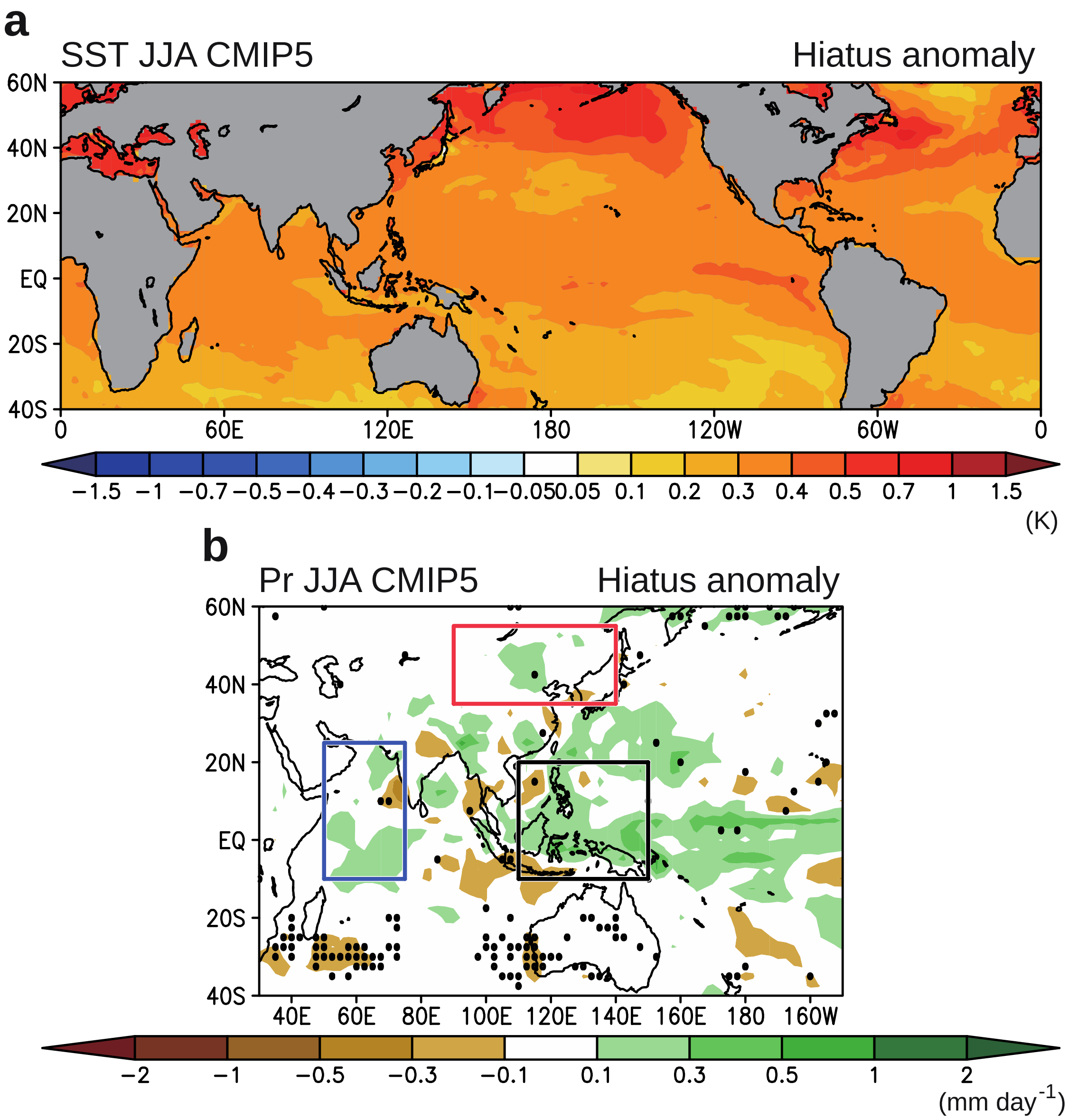

Supplementary Figure 2 | Simulated patterns in multiple climate models during hiatus. (a) SST (K) and (b) precipitation (mm day^{-1}). Anomalies are regime differences between periods 1999–2013 and 1979–1998 obtained from Coupled Model Intercomparison Project Phase 5 (CMIP5) multi-model archive. 26 models (Supplementary Table 1) were used for the analysis. Stipples in (b) denote regions where 22 out of 26 models agree on sign of the values.

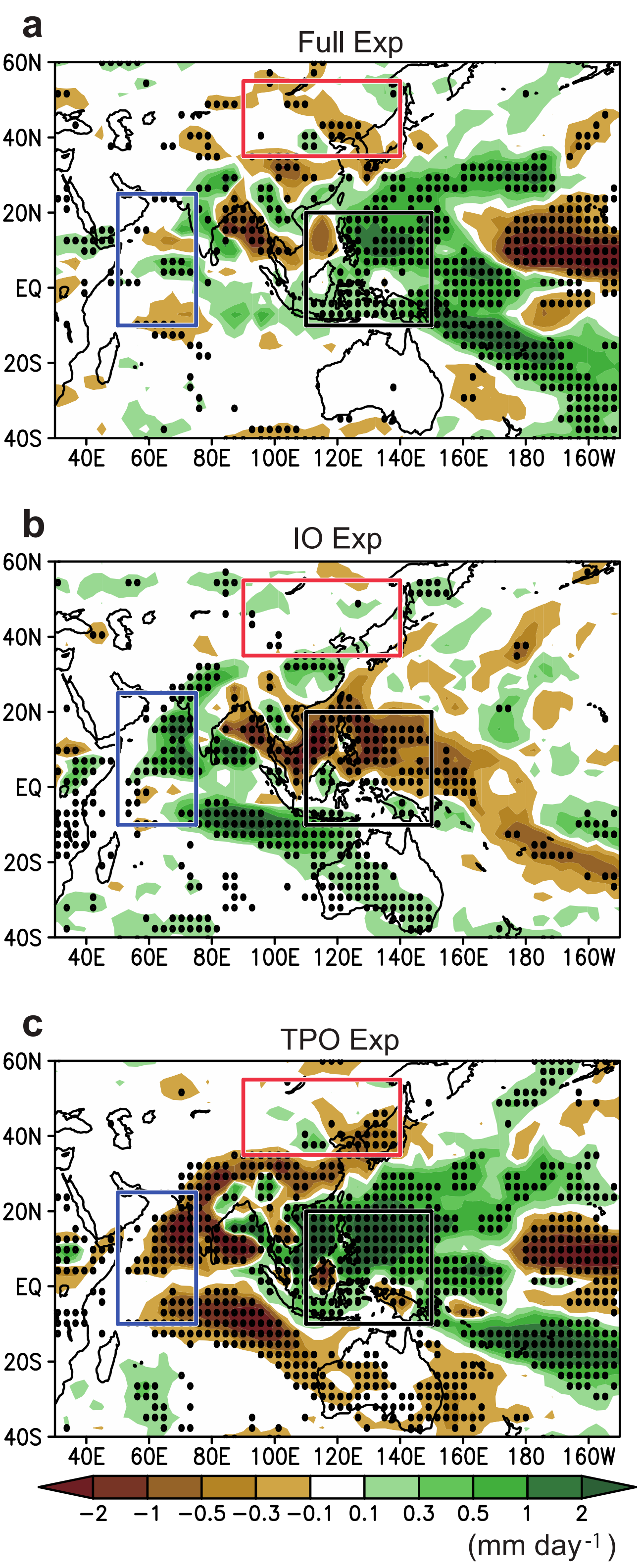

Supplementary Figure 3 | Statistical significance in the simulated rainfall. Shading is as in Fig. 3. Rainfall anomalies significant above the 95% confidence level are stippled.

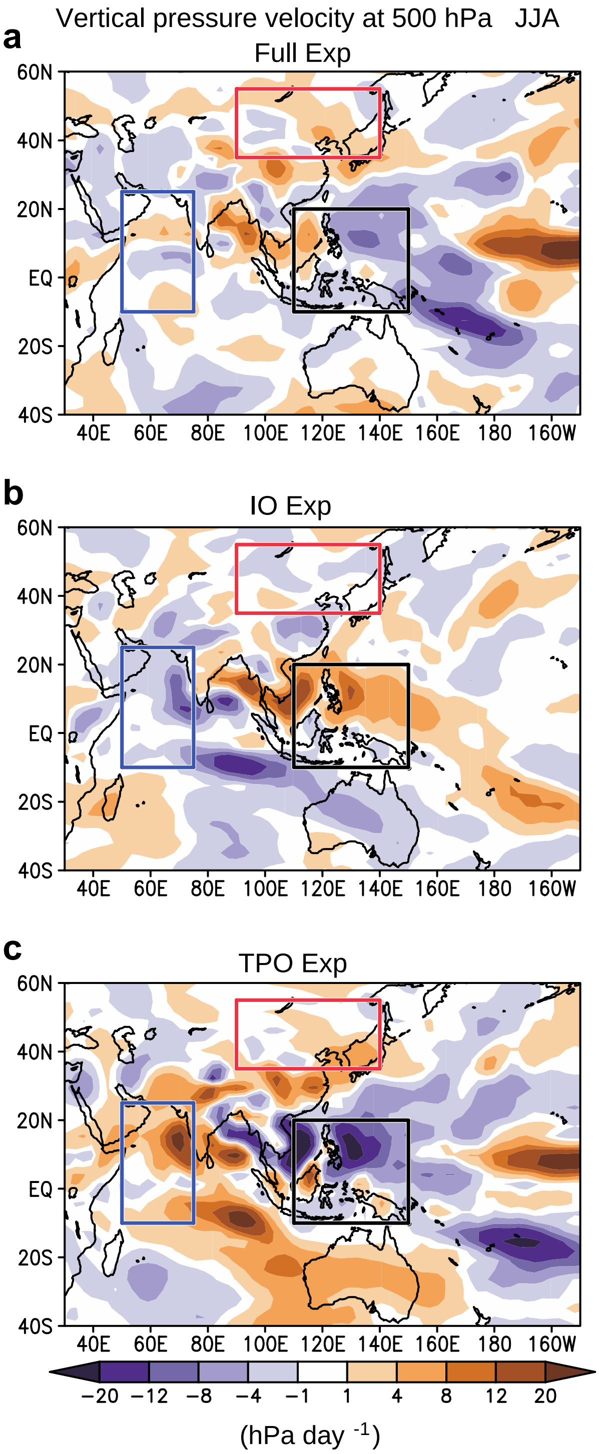

Supplementary Figure 4 | Simulated patterns of vertical motion for the boreal summer. **(a)** Omega (hPa day^{-1}) at 500 hPa from the MRI-AGCM 10 experiments, where SSTs were set to hiatus anomalies (1999–2013). **(b)** and **(c)** as for **(a)** but for the effects of the Indian Ocean and the tropical Pacific, respectively (see Methods). Red, blue, and black rectangles are the EA, WIO, and WP regions, respectively. Positive (negative) values indicate downward (upward) motion.

Supplementary Table 1 | List of the CMIP5 models used in Supplementary Figure 2.

Model name	Country
ACCESS1.0	Australia
ACCESS1.3	Australia
BCC-CSM1.1(m)	China
CCSM4	USA
CESM1(BGC)	USA
CESM1(CAM5)	USA
CMCC-CM	Italy
CNRM-CM5	France
CSIRO-Mk3-6-0	Australia
CanESM2	Canada
FGOALS-g2	China
FGOALS-s2	China
FIO-ESM	China
GFDL-CM3	USA
GFDL-ESM2G	USA
GISS-E2-H	USA
GISS-E2-R	USA
HadGEM2-ES	UK
INM-CM4	Russia
IPSL-CM5A-LR	France
MIROC-ESM	Japan
MIROC5	Japan
MPI-ESM-LR	Germany
MPI-ESM-MR	Germany
MRI-CGCM3	Japan
NorESM1-M	Norway

Supplementary Note 1

Anthropogenic influences on the tropical SST and Asian monsoon during the recent warming hiatus

As mentioned in the main body, several studies[1-3] showed that natural variability including PDO contributes considerably to the recent warming hiatus in which anthropogenic effect becomes smaller over shorter time scale[4] (~15 years). Regarding the future projection, direct effects of CO_2 on the atmospheric circulations over the tropics and mid-latitude are much weaker than the effect of sea surface temperature (SST) variation[5,6]. The latest review[7] for reduction of uncertainties on regional climate (greater than 100 km) suggests that understanding the tropical upper-ocean temperature is central importance when we try to narrow the uncertainty because the tropical atmospheric circulation is tightly associated with the variation in the SST pattern.

Returning to distribution of the tropical SST anomaly during the hiatus (Fig. 2a), it is conceivable that its shape and amplitude could not be ascribed to the anthropogenic forcing. To confirm the relative contribution of the anthropogenic effect and internal variability, we examined historical and future climate projections conducted under the Coupled Model Intercomparison Project Phase 5 (CMIP5)[8]. 26 models were used in the analyses. Benefit of use of the model ensemble is that the anthropogenic influence on the climate variation can be evaluated because the internal variability in each model is canceled in the ensemble mean.

Supplementary Fig. 2 shows result of historical runs (1979–2005) and future projections (representative concentration pathways 4.5; 2006–2013)[8]. Comparing to the observation (Fig. 1a and Fig. 2a), the simulated SST exhibits zonally-uniform warming (i.e. any cooling signals cannot be found in the Pacific) and resultant rainfall distribution also does not display noticeable and robust pattern over the Asian monsoon region. For example, precipitation changes over WP, WIO and EA regions are not robust across 26 models. In contrast, precipitation decline over the Southern Hemisphere middle latitude is found to be robust because of the possible influence from the expansion of the Hadley circulation due to the anthropogenic forcing[5]. On the contrary, if we prescribe the observed SST (Fig. 2a), the AGCM used in this study succeeded to reproduce the observed rainfall variation over the Asian monsoon region (Fig. 3a). Although the anthropogenic forcing is crucial factor for the surface warming over the Indian Ocean and Atlantic (Fig. 2a), the multi-model analysis shows that the anthropogenic influence cannot explain the precipitation anomaly over Asia solely. These results indicate that the natural variability is responsible for the recent unique patterns of SST and ensuing rainfall variations.

Supplementary References

1. Meehl, G. A., Arblaster, J. M., Fasullo, J. T., Hu, A. & Trenberth K. E. Model-based evidence of deep-ocean heat uptake during surface-temperature hiatus periods. *Nature Clim. Change* **1**, 360–364 (2011).
2. England, M. H. McGregor, S, Spence, P., Meehl, G. A., Timmermann, A., Cai, W., Gupta, A. S., McPhaden, M. J., Purich, A. & Santoso, A. Recent intensification of wind-driven circulation in the Pacific and the ongoing warming hiatus. *Nature Clim. Change* **4**, 222–227 (2014).
3. Kosaka, Y. & Xie, S-P. Recent global-warming hiatus tied to equatorial Pacific surface cooling. *Nature* 501, 403–407 (2013).
4. Watanabe, M., Shiogama, H., Tatebe, H., Hayashi, M., Ishii, M. & Kimoto, M. Contribution of natural decadal variability to global warming acceleration and hiatus. *Nature Clim. Change* **4**, 893–897 (2014).
5. Ma, J., Xie, S.-P. & Kosaka, Y. Mechanisms for tropical tropospheric circulation change in response to global warming. *J. Clim.* **25**, 2979–2994 (2012).
6. Grise, K.M. & Polvani, L.M. The response of midlatitude jets to increased CO_2: Distinguishing the roles of sea surface temperature and direct radiative forcing. *Geophys. Res. Lett.* **41**, 6863–6871 (2014).
7. Xie, S.-P. Deser, C., Vecchi, G.A., Collins, M., Delworth, T.L., Hall, A., Hawkins, E., Johnson, N.C., Cassou, C., Giannini, A. & Watanabe, M. Towards predictive understanding of regional climate change. *Nature Clim. Change* **5**, doi:10.1038/nclimate2689 (2015).
8. Taylor, K. E. et al. An overview of CMIP5 and the experiment design. *Bull. Am. Meteorol. Soc.* **93**, 485–498 (2012).
